# Efficient Skip Connections-Based Residual Network (ESRNet) for Brain Tumor Classification

**DOI:** 10.3390/diagnostics13203234

**Published:** 2023-10-17

**Authors:** Ashwini B., Manjit Kaur, Dilbag Singh, Satyabrata Roy, Mohammed Amoon

**Affiliations:** 1 Department of ISE, NMAM Institute of Technology, Nitte (Deemed to be University), Nitte 574110, India; ashwinib@nitte.edu.in; 2 School of Computer Science and Artificial Intelligence, SR University, Warangal 506371, India; 3 Center of Biomedical Imaging, Department of Radiology, New York University Grossman School of Medicine, New York, NY 10016, USA; dggill2@gmail.com; 4 Research and Development Cell, Lovely Professional University, Phagwara 144411, India; 5 Department of Computer Science and Engineering, Manipal University Jaipur, Jaipur 303007, India; satyabrata.roy@jaipur.manipal.edu; 6 Department of Computer Science, Community College, King Saud University, P.O. Box 28095, Riyadh 11437, Saudi Arabia

**Keywords:** brain tumor classification, deep learning, residual networks, vanishing gradient, feature learning, medical diagnostics

## Abstract

Brain tumors pose a complex and urgent challenge in medical diagnostics, requiring precise and timely classification due to their diverse characteristics and potentially life-threatening consequences. While existing deep learning (DL)-based brain tumor classification (BTC) models have shown significant progress, they encounter limitations like restricted depth, vanishing gradient issues, and difficulties in capturing intricate features. To address these challenges, this paper proposes an efficient skip connections-based residual network (ESRNet). leveraging the residual network (ResNet) with skip connections. ESRNet ensures smooth gradient flow during training, mitigating the vanishing gradient problem. Additionally, the ESRNet architecture includes multiple stages with increasing numbers of residual blocks for improved feature learning and pattern recognition. ESRNet utilizes residual blocks from the ResNet architecture, featuring skip connections that enable identity mapping. Through direct addition of the input tensor to the convolutional layer output within each block, skip connections preserve the gradient flow. This mechanism prevents vanishing gradients, ensuring effective information propagation across network layers during training. Furthermore, ESRNet integrates efficient downsampling techniques and stabilizing batch normalization layers, which collectively contribute to its robust and reliable performance. Extensive experimental results reveal that ESRNet significantly outperforms other approaches in terms of accuracy, sensitivity, specificity, F-score, and Kappa statistics, with median values of 99.62%, 99.68%, 99.89%, 99.47%, and 99.42%, respectively. Moreover, the achieved minimum performance metrics, including accuracy (99.34%), sensitivity (99.47%), specificity (99.79%), F-score (99.04%), and Kappa statistics (99.21%), underscore the exceptional effectiveness of ESRNet for BTC. Therefore, the proposed ESRNet showcases exceptional performance and efficiency in BTC, holding the potential to revolutionize clinical diagnosis and treatment planning.

## 1. Introduction

Brain tumors (BT) represent a multifaceted and critical challenge within the field of medical diagnostics. The diverse characteristics and potentially life-threatening consequences of these tumors demand precise and timely classification [1]. As a leading cause of morbidity and mortality globally, the imperative for advanced diagnostic tools and methodologies in brain tumor classification (BTC) cannot be overstated. Accurate BTC is a multifaceted challenge, requiring the capability to differentiate between various tumor types, each characterized by its unique morphological, genetic, and clinical features [2]. The significance of accurate classification is far-reaching—it ensures timely treatment, optimizes patient care, and, thus, improves survival rates. Traditional diagnostic methods often rely on subjective interpretations by radiologists, introducing variability in accuracy and potentially delaying critical diagnoses.

In recent years, DL has revolutionized BTC in medical imaging [3]. Its ability to autonomously discern intricate patterns from vast datasets holds great potential for addressing these challenges [4,5]. Togacar et al. [1] introduced innovative DL models, notably BrainMRNet, incorporating attention modules, the hypercolumn technique, and residual blocks to achieve remarkable classification accuracy for glioma, meningioma, and pituitary tumors. Similarly, Hashmi and Osman [2] explored BTC using residual networks and an attention approach, demonstrating substantial accuracy improvements. Furthermore, Papadomanolakis et al. [3] presented a novel diagnostic framework based on convolutional neural networks (CNNs) and discrete wavelet transform (DWT) data analysis for glioma tumor diagnosis, showcasing impressive performance with potential clinical applications. Lastly, Mahum et al. [6] proposed an effective approach that utilizes feature fusion, leveraging the mayfly optimization algorithm and multilevel thresholding for tumor localization. Their bidirectional long short-term memory (BiLSTM) network achieved remarkable results in classifying pituitary, glioma, and meningioma tumors.

Amou et al. [7] introduced a pioneering Bayesian optimization-based technique to optimize the hyperparameters for CNNs, resulting in outstanding accuracy in the classification of brain tumors from MRI images. Additionally, Sunsahi [8] developed the adaptive eroded deep CNN (AEDCNN), showcasing its effectiveness in the segmentation and classification of brain images, identifying meningioma, glioma, and pituitary tumors. Rizwan et al. [9] presented a Gaussian CNN (GCNN) that achieved exceptional accuracy in classifying brain tumors and differentiating glioma grades in a multi-class context. Furthermore, Kothandaraman [10] harnessed the binary swallow swarm optimization to augment the performance of CNNs for BTC, offering a promising avenue for automating tumor detection. Lastly, Chitnis et al. [11] introduced the learning-by-self-explanation (LeaSE) architecture search method, automating the discovery of high-performance neural architectures for BTC. This approach outperformed manually designed networks in both accuracy and parameter efficiency.

Existing DL techniques have demonstrated substantial advancements in enhancing the accuracy and efficiency of BTC. These developments hold the promise of delivering more precise and timely diagnoses, thereby bolstering the quality of patient care. Automation plays a pivotal role by diminishing the dependence on human interpretation, a factor that can lead to a reduction in errors. This, in turn, contributes to an overall enhancement in the quality of medical care provided to patients. Furthermore, the incorporation of advanced techniques, such as attention modules and segmentation methods, facilitates superior feature extraction. This heightened capability enables the discernment of intricate and nuanced tumor characteristics, thereby amplifying the diagnostic potential of these technologies.

This paper introduces a pioneering approach to tackle the challenges associated with accurate and reliable BTC by proposing an efficient skip connections-based residual network (ESRNet). With the ever-growing complexity of medical data, particularly in the realm of brain imaging, traditional models often face limitations such as in-depth, gradient flow, and feature extraction [12]. These limitations often result in models that struggle to learn and represent the underlying complexities of brain tumor images adequately. Limited depth can hinder the model’s capacity to extract hierarchical features, potentially causing the network to miss critical patterns and details within the data. Additionally, vanishing gradient problems can impede the training process, making it challenging to optimize deep networks effectively. Furthermore, intricate features, which are essential for accurate tumor classification, may not be well-captured by shallower architectures, leading to suboptimal performance.

The proposed ESRNet utilizes residual blocks from the ResNet architecture, featuring skip connections that enable identity mapping. Through direct addition of the input tensor to the convolutional layer output within each block, skip connections preserve the gradient flow. This mechanism prevents vanishing gradients, ensuring effective information propagation across network layers during training. Thus, the proposed architecture ensures smooth gradient flow during training, mitigating the vanishing gradient problem and facilitating the learning of intricate features. The strategic incorporation of efficient downsampling techniques and batch normalization further enhances computational efficiency. ESRNet’s unique design, organized into stages with increasing numbers of residual blocks, promotes in-depth feature learning, setting the stage for a model that not only outperforms existing benchmarks but also holds the potential to revolutionize brain tumor classification in clinical settings. Feature learning refers to the process in machine learning where a model automatically learns to represent relevant features from raw data, and it is not specifically related to feature selection. This paper makes the following key contributions:

1. ESRNet: The efficient skip connections-based residual network (ESRNet) is proposed for BTC.
2. Residual Blocks with Skip Connections: The proposed ESRNet incorporates residual blocks with skip connections, enabling the construction of deep neural networks. These skip connections effectively mitigate the vanishing gradient problem, facilitating deep network training and enhancing the gradient flow.
3. Increased Depth and Enhanced Feature Learning: The proposed ESRNet is organized into five stages, each progressively incorporating more residual blocks. This increased depth enhances ESRNet’s capacity for in-depth feature learning, enabling the capture of intricate patterns and significantly improving classification performance.
4. Efficient Downsampling and Batch Normalization: The architecture of ESRNet includes efficient downsampling at specific stages while maintaining computational efficiency. Batch normalization layers are seamlessly integrated into the residual blocks to stabilize and expedite training, contributing to the overall efficiency and performance of BTC.

The remainder of the paper is organized into the following sections: Section 2 provides an overview of related work in the field. Section 3 introduces the proposed efficient skip connections-based residual network (ESRNet). Section 4 details the experimental setup and presents comparative results. Finally, Section 5 presents the conclusions and summarizes the key findings of the paper.

## 2. Related Work

In recent years, significant progress has been achieved in the field of BTC using DL and machine learning (ML) techniques. Several studies have explored various approaches to enhance the accuracy and efficiency of brain tumor detection and classification. The following related work highlights key contributions in this area.

Qureshi et al. proposed an intelligent ultra-light DL model for multi-class brain tumor detection [13]. The approach leveraged an ultra-light DL architecture, integrated with distinctive textural features extracted using the gray-level co-occurrence matrix (GLCM). This hybrid feature space was then used for tumor detection with support vector machine (SVM), achieving high prediction accuracy. Saha et al. introduced the BCM-VEMT system, which combined DL and an ensemble of ML techniques for BTC [14]. The system achieved high accuracy in classifying different brain tumor types. The approach is valuable for aiding medical decisions.

Kibriya et al. presented a CNN architecture for multiclass BTC [15]. Their 13-layer CNN achieved superior accuracy, outperforming previous work on benchmark datasets. The lightweight architecture facilitated rapid tumor detection, aiding early-stage diagnosis. Yazdan et al. proposed an efficient multi-scale CNN for multi-class brain MRI classification [16]. Their model addressed challenges related to Rician noise and achieved high accuracy. The proposed architecture outperformed other DL models, making it suitable for clinical research. Sekhar et al. introduced a BTC system using fine-tuned GoogLeNet features and ML algorithms [17]. Their IoMT-enabled CAD system demonstrated the potential to detect and classify tumors accurately. The approach was found to be valuable for early diagnosis and remote healthcare.

Ahmad et al. devised a novel method for BTC [18]. They introduced a framework merging variational autoencoders (VAEs) and generative adversarial networks (GANs) to tackle limited medical image datasets. Their approach generated artificial MRI images, significantly elevating accuracy from 72.63% to 96.25%. Zulfiqar et al. employed EfficientNets for multi-class BTC [19]. Through transfer-learning-based fine-tuning and data augmentation, they attained remarkable results with an overall test accuracy of 98.86%, underlining the efficacy of DL models. Demir and Akbulut introduced a novel DL technique for the brain MRI classification [20]. Their residual-CNN (R-CNN) model, complemented by L1NSR feature selection, achieved high classification accuracies of 98.8% for 2-class and 96.6% for 4-class datasets, demonstrating the potential of DL in precise tumor classification.

Zahid et al. [21] designed BrainNet, an efficient deep learning model for optimal feature fusion in BTC. By leveraging advanced neural network architectures, the authors aimed to enhance the accuracy of BTC. The proposed BrainNet showcased the challenges associated with brain tumor analysis, marking a notable advancement in the application of deep learning for medical image classification. Maqsood et al. [22] presented TTCNN as a deep learning model tailored for breast cancer detection and classification using digital mammography. Emphasizing early-stage diagnosis, TTCNN underscored the potential impact of computer-aided diagnosis methods in breast cancer detection. Raza et al. [23] introduced DeepTumorNet, a hybrid model for BTC, integrating traditional CNNs with tailored modifications to the GoogLeNet architecture. The strategic customization, including the removal of the last five layers and the addition of 15 new layers, demonstrated a nuanced understanding of BTC intricacies.

Vankdothu et al. [24] introduced a brain tumor identification and classification method based on a CNN-LSTM architecture. The layered CNN design demonstrated superior performance in image classification compared to standard CNN-LSTM approaches. Experimental findings revealed that the proposed model outperformed earlier CNN and RNN models in terms of accuracy. Maqsood et al. [25] proposed a multi-modal brain tumor detection method. The approach involved linear contrast stretching, a custom 17-layered neural network for segmentation, modified MobileNetV2 for feature extraction, and an entropy-based method coupled with M-SVM for optimal feature selection. The final step employed M-SVM for accurate BTC, identifying meningioma, glioma, and pituitary images.

Mohammad et al. pioneered a blockchain-based deep CNN model for MRI-based brain tumor prediction [26], offering enhanced security and precision in tumor prediction, showing the promise of blockchain in medical imaging. Reza et al. devised an efficient CNN-based strategy for classifying MRI-based tumors [27]. Their modified VGG-16 architecture yielded exceptional precision and accuracy, with 99.4% for glioma, 96.7% for meningioma, 100% for pituitary tumors, and an overall accuracy of 99.5%, affirming the significance of DL models in precise tumor classification. El-Wahab et al. introduced BTC-fCNN, a fast and efficient DL-based system for multi-class BTC. They achieved an average accuracy of 98.63% using transfer learning and 98.86% with retrained five-fold cross-validation, surpassing state-of-the-art methods [28]. Maqsood, Damasevicius, and Maskeliunas presented a multi-modal brain tumor detection method using deep neural networks and multiclass SVM. Their approach achieved an accuracy of 97.47% for detection and 98.92% for classification, outperforming other methods [25].

Gupta et al. proposed a brain tumor detection and classification system. They used an ensemble approach combining modified InceptionResNetV2 and Random Forest Tree to achieve 99% accuracy for tumor detection and 98% for classification [29]. Oksuz et al. introduced a BTC method using fused features extracted from expanded tumor regions. By fusing deep and shallow features, they improved the sensitivity by approximately 11.72%. Their approach leveraged deep networks, like AlexNet and ResNet-18 [30]. Kesav and Jibukumar proposed an efficient and low-complexity architecture for brain tumor detection and classification. They used a two-channel CNN and RCNN, achieving an accuracy of 98.21% for classification and low execution times, outperforming complex architectures [31].

Rasheed et al. introduced a CNN model for BTC. Their method achieved a remarkable classification accuracy of 98.04% for glioma, meningioma, and pituitary tumors. This algorithm demonstrated superior performance compared to existing pre-trained CNN models [32]. Polat and Gungen proposed a solution using transfer learning with networks like VGG16, VGG19, ResNet50, and DenseNet21. Their model achieved a high classification performance of 99.02%, particularly with ResNet50 using the Adadelta optimization algorithm [33]. Alanazi et al. introduced a novel transfer-deep-learning model for BTC into subclasses. This model achieved an accuracy of 95.75% for MRI images from the same machine and demonstrated adaptability to different MRI machines, showcasing its potential for real-time application [34].

Al-Zoghby et al. developed a dual CNN model for classifying three types of brain tumors. Their model reached a remarkable accuracy of 100% during training and 99% during testing, showcasing significant improvements over existing research [35]. Rehman et al. conducted comprehensive studies using CNN models (VGGNet, GoogLeNet, and AlexNet) for BTC. The fine-tuned VGG16 architecture achieved the highest accuracy, up to 98.69%, for the classification and detection of brain tumors [36]. Vankdothu et al. proposed an IoT computational system based on DL for brain tumor detection in MRI images. Their LSTM-CNN model outperformed standard CNN classification and showed improved accuracy in detecting brain tumors [24].

Mahmoud et al. trained CNN models for detecting the most prevalent brain tumor types and achieved an impressive accuracy of 98.95%, particularly with the VGG-19 model [37]. Diaz-Pernas et al. presented a BTC model using a multiscale CNN. Their model achieved a tumor classification accuracy of 97.3%, outperforming other methods on the same dataset [38]. Anjum et al. compared DL methods with transfer learning to traditional ML techniques for brain tumor detection. DL methods, especially those based on ResNet101 with transfer learning, demonstrated superior performance and a promising potential for prognosis and treatment planning [39].

In summary, the current DL models designed for BTC encounter a range of formidable challenges. These include issues related to limited network depth, the potential occurrence of vanishing gradient problems, and the complexities associated with capturing intricate image features. These constraints collectively contribute to the models’ struggles in effectively learning and representing the underlying intricacies present within brain tumor images. The restricted depth of these models can hinder their ability to extract hierarchical features, which, in turn, may lead to crucial patterns and image details being overlooked during the analysis. Furthermore, the presence of vanishing gradient problems can disrupt the training process, posing difficulties in achieving optimal performance when dealing with deep networks. Moreover, shallower architectures might struggle to adequately capture the intricate features crucial for precise tumor classification, resulting in suboptimal model performance.

## 3. Efficient Skip Connections-Based Residual Network (ESRNet)

Inspired by [12], this paper proposes a comprehensive enhancement to BTC through an ESRNet. ESRNet incorporates residual blocks with skip connections, facilitating the training of deep neural networks by mitigating the vanishing gradient problem (see Algorithm 1). ESRNet is structured into five stages, each progressively integrating more residual blocks, leading to improved feature learning and the ability to capture intricate patterns. Furthermore, the architecture of ESRNet incorporates efficient downsampling techniques and batch normalization layers, optimizing computational efficiency while stabilizing and expediting the training process. In the following section, we present the architecture of a proposed ESRNet for the classification of brain tumors.
**Algorithm 1:** Efficient Skip Connections-based Residual Network (ESRNet)
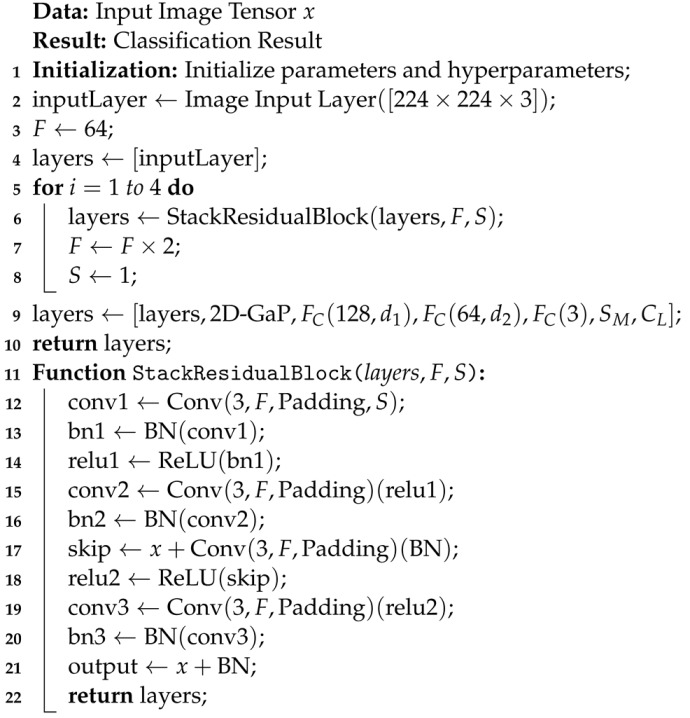


### 3.1. Residual Block with Convolution Layers

A residual block is a fundamental building block of the ResNet architecture. It consists of multiple convolutional layers (Conv), batch normalization (BN), skip connections (Skip), and the addition operation (Add). The formula for a residual block can be expressed as follows:

(1) $$\begin{array}{cc} \mathrm{function}\;\mathrm{layers} & =\mathrm{residualBlock}(x,F,S)\\ \; & =\left[\begin{array}{c} \mathrm{Conv}(3,F,\mathrm{Padding},S)(x)\\ \mathrm{BN}(\mathrm{Conv})(x)\\ \mathrm{ReLU}(\mathrm{BN})\\ \mathrm{Conv}(3,F,\mathrm{Padding})(\mathrm{ReLU})\\ \mathrm{BN}(\mathrm{Conv})\\ \mathrm{Skip}:x+\mathrm{Conv}(3,F,\mathrm{Padding})(\mathrm{BN})\\ \mathrm{ReLU}(\mathrm{Skip})\\ \mathrm{Conv}(3,F,\mathrm{Padding})(\mathrm{ReLU})\\ \mathrm{BN}(\mathrm{Conv})\\ \mathrm{Final}\;\mathrm{Output}:x+\mathrm{BN} \end{array}\right] \end{array}$$

where *x* denotes the input tensor, *F* signifies the number of filters employed within the convolutional layers, and *S* represents the stride utilized in these convolutional layers. Additionally, ‘Conv’ stands for the convolutional layer, ‘BN’ indicates the batch normalization layer, ‘ReLU’ signifies the rectified linear unit activation function, ‘Skip’ represents the skip connection, which performs the identity mapping, and ‘Add’ denotes the operation of element-wise addition. However, the ‘Add’ operation is not explicitly utilized; instead, the ‘Skip’ connection is employed, representing the essence of an addition operation (element-wise addition) between *x* and the outcome of a ‘Conv’ followed by ‘BN’.

### 3.2. Stage 1: Building Depth and Feature Learning

The construction of ESRNet involves a process of stacking multiple residual blocks, which are essential for enhancing the network’s depth and feature-learning capabilities. This architectural design is organized into four distinct stages, each progressively increasing the number of residual blocks within. Crucially, skip connections are meticulously maintained between these stages to ensure a smooth gradient flow during training. To offer a clearer view of ESRNet’s foundational structure, consider the following equation:

(2) $$\begin{array}{cc} \mathrm{inputLayer} & =\mathrm{Image}\;\mathrm{Input}\;\mathrm{Layer}([224\;224\;3])\\ \mathrm{layers} & =\left[\begin{array}{c} \mathrm{inputLayer}\\ \mathrm{Conv}(7,F,\mathrm{Padding},S)\\ \mathrm{BN}(\mathrm{Conv})\\ \mathrm{ReLU}(\mathrm{BN})\\ \mathrm{MaxPooling}(3,S) \end{array}\right] \end{array}$$

At the initial stage, we begin with an input layer designed to accommodate image data with dimensions of 224×224×3. The number of filters, denoted as *F*, is set to 64. Within this stage, we sequentially stack several essential layers, including a convolutional layer with kernel size 7, batch normalization following the convolution, rectified linear unit (ReLU) activation, and a max-pooling layer with a kernel size of 3 and an appropriate stride (*S*). These operations serve to progressively extract and process features from the input data. This initial stage lays the foundation for the subsequent stages, collectively forming the ResNet model’s robust architecture.

### 3.3. Stage 2: Stack Three Residual Blocks with Skip Connections

In the second stage of ESRNet, we stack three residual blocks with skip connections. The stride of the first block is set to 2 to downsample the feature maps. It can be defined as follows:

(3) $$S=\left\{\begin{array}{cc} 1 & \mathrm{for}\;i=1\\ 2 & \mathrm{for}\;i>1 \end{array}\right.$$

Here, the stride value *S* alternates between 1 and 2 based on the iteration index *i*, allowing for downsampling in the initial block and maintaining the stride at 1 for subsequent blocks. These changes in stride are utilized when stacking the three residual blocks, effectively controlling the feature map size in the second stage.

### 3.4. Stage 3: Capture Intricate Features

In Stage 3, we further enhance ESRNet’s capacity to capture intricate features. This stage builds upon the foundation laid in Stage 2 with some notable differences. Firstly, we double the number of filters (*F*) compared to Stage 2, allowing ESRNet to explore more complex patterns and representations. Secondly, as in Stage 2, the first residual block initiates with a stride of 2 to downsample the feature maps, ensuring spatial reduction. However, in contrast to Stage 2, where all subsequent residual blocks maintain a stride of 1, in Stage 3, we continue with a stride of 1 throughout. This strategic choice preserves the spatial dimensions of feature maps for the remainder of this stage. These modifications between Stage 2 and Stage 3 contribute to ESRNet’s progressive feature learning, enhancing its capability to classify brain tumors effectively.

### 3.5. Stage 4: High-Level Abstractions

In Stage 4, we continue to deepen ESRNet while introducing specific changes compared to Stage 3. Similar to the previous stage, we double the number of filters (*F*), enabling ESRNet to capture even more intricate features and representations. However, the key difference lies in how we downsample the feature maps. While in Stage 3, the first block had a stride of 2 for downsampling, in Stage 4, we maintained this stride of 2 for the first block to reduce the spatial dimensions effectively. This choice allows ESRNet to focus on high-level abstractions by reducing the spatial resolution. Furthermore, we stack six residual blocks in Stage 4, compared to four in Stage 3, further enhancing ESRNet’s capacity to learn complex features. These alterations between Stage 3 and Stage 4 contribute to ESRNet’s increasing depth and representational power, making it more capable of classifying brain tumors accurately.

### 3.6. Stage 5: Enhanced Depth and Feature Learning

In the fifth and final stage, we maintain the architectural pattern established in the previous stages while introducing specific changes to adapt to the increasing depth. Similar to Stage 4, we double the number of filters (*F*), allowing ESRNet to capture high-level features effectively. However, the critical alteration lies in the stride value (*S*) for downsampling. In this stage, as in Stage 4, the first block employs a stride of 2 to reduce the spatial dimensions of the feature maps, enhancing the network’s focus on more abstract representations. Subsequently, we stack three residual blocks, maintaining the same pattern as in previous stages. This stage’s adjustments, specifically the increase in filter count and the strategic use of stride for downsampling, contribute to ESRNet’s enhanced depth and feature learning, making it well-suited for precise BTC.

### 3.7. Final Layers

The final layers of ESRNet include a global average pooling layer followed by fully connected layers, each integrated with dropout for regularization. The architecture concludes with a softmax activation layer and a classification layer.

(4) $$\begin{array}{cc} \mathrm{layers} & =\left[\begin{array}{c} \mathrm{layers}\\ 2D-\mathrm{GaP}\\ F_{C}\left(128,d_{1}\right)\\ F_{C}\left(64,d_{2}\right)\\ F_{C}\left(3\right)\\ S_{M}\\ C_{L} \end{array}\right] \end{array}$$

Here, the 2D−GaP layer plays a crucial role in global feature extraction by performing global average pooling on the feature maps. Two pivotal fully connected ($F_{C}$) layers, namely $F_{C}\left(512,d_{1}\right)$ and $F_{C}\left(256,d_{2}\right)$, are strategically inserted in the network. The former boasts 128 units and incorporates a dropout mechanism with a rate of $d_{1}$ for regularization, while the latter consists of 64 units and employs dropout with a rate of $d_{2}$ to enhance model generalization. The architecture culminates with a $F_{C}\left(3\right)$, equipped with three output units to represent the three distinct tumor classes. Subsequently, the $S_{M}$ layer applies softmax activation to calculate probability distributions, while the final classification decision is determined by the $C_{L}$ layer, which assigns the input data to one of the tumor classes based on the softmax probabilities. This intricate arrangement of layers and components collectively forms a robust and efficient framework for accurate BTC.

### 3.8. Sparse Categorical Cross-Entropy Loss

In the training process of ESRNet for BTC, we employ the sparse categorical cross-entropy (SCCE) loss as the chosen loss function. This loss function is well-suited for multi-class classification tasks, particularly when class labels are represented as integers instead of one-hot encoded vectors.

The SCCE loss measures the dissimilarity between the predicted class probabilities generated by the model and the actual integer class labels of the input data samples. It effectively guides the training process by quantifying the error between the predictions ($\hat{y}$) and the ground truth labels (*y*), facilitating the optimization of the neural network’s parameters to achieve accurate classification results. Mathematically, SCCE can be defined as:

(5) $$L\left(y,\hat{y}\right)=-\frac{1}{N}\sum_{i=1}^{N}\sum_{j=1}^{C}\left(1\left\{y_{i}=j\right\}\cdot \log\left({\hat{y}}_{ij}\right)\right)$$

where $L(y,\hat{y})$ represents the loss function, *N* is the number of training samples, *C* is the number of classes (in our case, 3 for meningioma, glioma, and pituitary tumor), $y_{i}$ denotes the true class label for the *i*th sample, ${\hat{y}}_{ij}$ represents the predicted probability of the *i*th sample belonging to class *j*, and $1\{y_{i}=j\}$ is an indicator function that equals 1 when $y_{i}$ is equal to *j*, and 0 otherwise.

The use of this loss function is a crucial component of ESRNet’s training pipeline, ensuring that the model learns to make informed and precise predictions for classifying brain tumors into distinct categories, including meningioma, glioma, and pituitary tumor.

### 3.9. Training Process

Algorithm 2 presents a training procedure for ESRNet utilizing the Adam optimizer. It takes essential inputs, such as the training data, learning rate, batch size, and the number of training epochs. During each epoch, the training data is shuffled and processed in mini-batches. The algorithm computes gradients of the loss function concerning the model parameters for each mini-batch. It utilizes the Adam optimization method to update these parameters, incorporating the first and second moments of the gradients. These moments are corrected for bias, and the model parameters are updated accordingly. This iterative process repeats for the specified number of epochs, ultimately resulting in trained ESRNet parameters.
**Algorithm 2:** Training Algorithm with Adam Optimizer and SCCE Loss
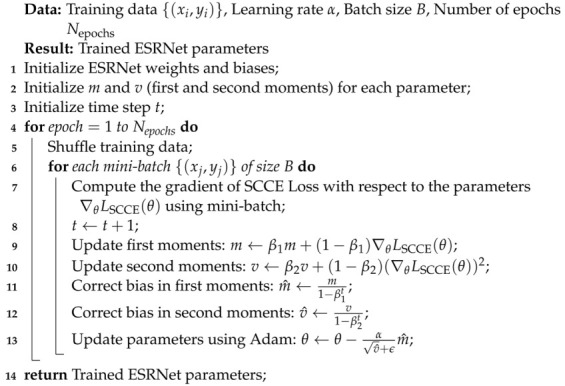


### 3.10. Hyperparameters

Table 1 presents the hyperparameters for ESRNet. These hyperparameters include the learning rate (α) for parameter updates, the batch size (*B*) determining the number of samples per mini-batch, and the total number of training epochs ($N_{\mathrm{epochs}}$). Additionally, the filter count (*F*) represents the number of filters in the convolutional layers, while two dropout rates ($d_{1}$ and $d_{2}$) control the probability of neuron dropout. The stride (*S*) defines the step size in the convolutional layers, and two decay rates ($\beta_{1}$ and $\beta_{2}$) influence the decay of the moment estimates in the Adam optimizer. The smoothing term (ϵ) is used in Adam optimization. The kernel size specifies the size of the convolution kernels, and the padding determines the type of padding applied.

## 4. Performance Analysis

The experiments were performed on MATLAB 2023a.

The computing platform was equipped with an 11th generation Intel^®^ Core™ i9-11950H vPro^®^ Processor, with a base clock speed of 2.60 GHz and a maximum turbo frequency of 5.00 GHz. A NVIDIA^®^ RTX™ A4000 Laptop GPU with 8 GB of GDDR6 graphics memory was used for accelerated processing. The memory capacity included 32 GB of DDR4-3200MHz SODIMM RAM, arranged as 2 × 16 GB modules, facilitating efficient data handling and processing during the experiments. The proposed ESRNet and competitive models including CNN [15], multi-scale CNN [16], ResNet-18 [30], CNN and RCNN [31], VAE and GAN [18], EfficientNets [19], BTC-fCNN [28], InceptionResNetV2 [29], modified VGG-16 [27], R-CNN [20], and fine-tuned GoogLeNet [17] were implemented for better comparative analysis. The hyperparameters of all the existing models were selected as reported in their respective research articles.

### 4.1. Dataset

**Figure 1:**
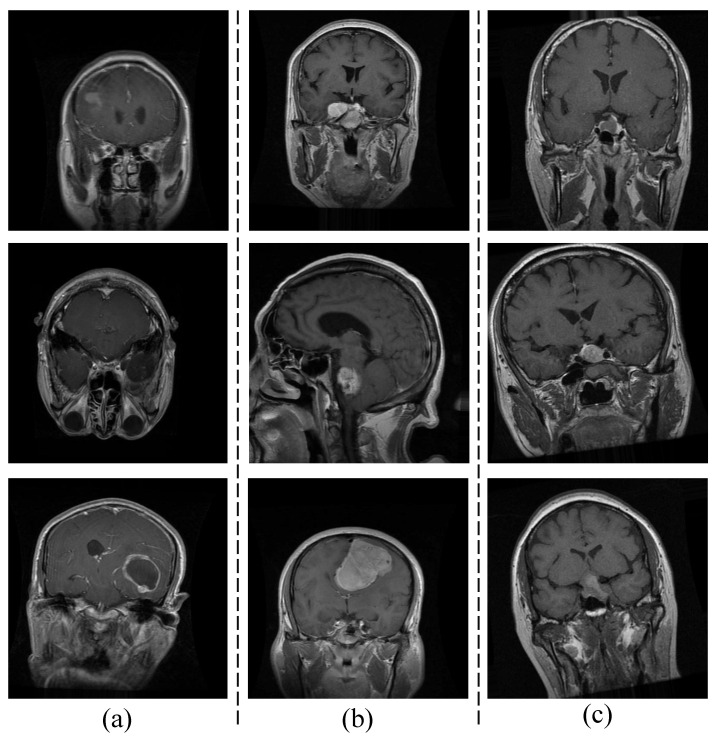
Sample images: (**a**) Glioma, (**b**) Meningioma, and (**c**) Pituitary Tumor.

Initially, the dataset consisted of 3064 T1-weighted, contrast-enhanced images derived from 233 patients who presented with three distinct types of brain tumors: meningioma (comprising 708 slices), glioma (comprising 1426 slices), and pituitary tumors (comprising 930 slices) [40]. Obuli [41] meticulously compiled this dataset, ensuring that each category contained 5000 images. Figure 1 displays sample images representing three distinct brain tumor types—(a) glioma, (b) meningioma, and (c) pituitary tumor—which were obtained from the dataset compiled by Obuli [41].

The dataset was further divided into three fractions, i.e., training, validation, and testing. The majority of the data (75%) was used for ESRNet’s training. This larger portion allows ESRNet to learn patterns and relationships in the data effectively. It is essential for training a model with sufficient capacity to capture complex patterns in the data. The validation dataset (10%) was used during the training process to monitor performance and tune the hyperparameters of ESRNet. It helped to prevent overfitting by allowing checking of how well ESRNet generalized to unseen data that it was not explicitly trained on. It was crucial for selecting the best model and hyperparameters. The remaining 15% was reserved for testing the performance of ESRNet. This set of data was entirely independent of both the training and validation sets. It provided an unbiased evaluation of ESRNet’s ability to generalize to new and unseen data.

The choice of the training, validation, and testing ratios was determined through a systematic experimentation process, considering a range of values for the training data fraction, spanning from 50% to 90%. The goal was to identify a configuration that optimally balanced model performance, generalization, and effective hyperparameter tuning. Following this exploration, it was observed that allocating 75% of the data to training yielded the most generalized and robust results for the proposed model. This particular ratio facilitated the model in learning intricate patterns and relationships within the data effectively, resulting in improved overall performance. The validation dataset, comprising 10%, was deemed sufficient for fine-tuning the hyperparameters during the training process without overly relying on a small subset. The remaining 15% allocated to testing ensured a comprehensive evaluation of the model’s generalization to previously unseen data. Thus, the selected ratios of 75% for training, 10% for validation, and 15% for testing were determined to be optimal through empirical experimentation.

### 4.2. Ablation Study

**Figure 2:**
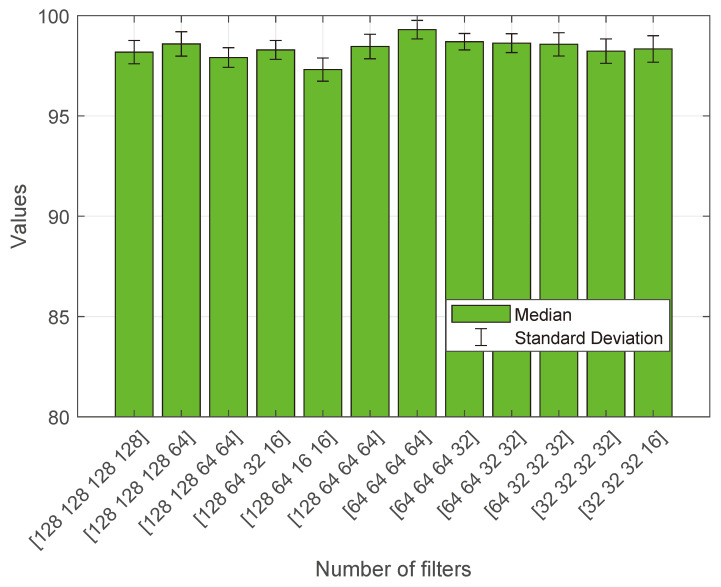
Number of filters analysis of ESRNet in terms of accuracy.

Figure 2 depicts an analysis of ESRNet’s performance in terms of accuracy based on varying numbers of filters. The experiment involved different filter configurations, such as ‘[128 128 128 128]’, ‘[128 128 128 64]’, and others. The results highlight that ESRNet attained exceptional performance, particularly when utilizing filters with the configuration ‘[64 64 64 64]’. This configuration yielded a remarkable accuracy of 99.62%±0.28%, showcasing the efficacy of this specific filter arrangement in optimizing model performance.

### 4.3. Loss Analysis

**Figure 3:**
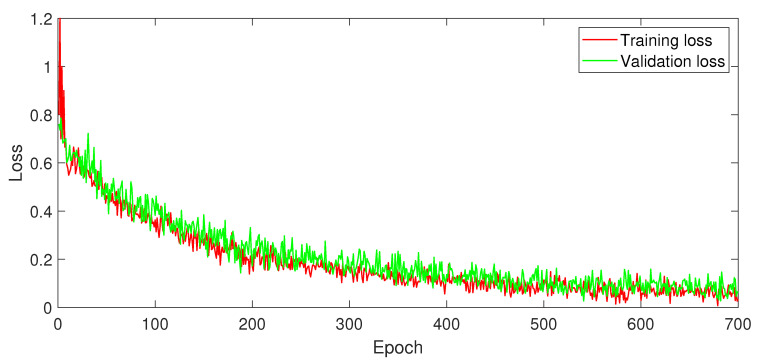
Loss analysis of ESRNet.

Figure 3 provides a loss analysis of ESRNet. The horizontal axis represents the number of training epochs. On the vertical axis, the loss values are presented, which measure how well ESRNet learned the data. The blue curve represents the training loss, indicating how well ESRNet fitted the training data over successive epochs. The orange curve represents the validation loss, measuring how well ESRNet generalized to new and unseen data. The smaller difference observed between the training and validation loss indicates a significantly lower impact of overfitting; thus, ESRNet can effectively generalize to real-world data.

The observed loss values approaching zero signify higher performance of ESRNet. These lower loss values indicate that ESRNet accurately captures the underlying patterns in the data. Additionally, these loss values indicate a better convergence speed during the training process, implying that ESRNet quickly achieves a better performance.

### 4.4. Confusion Matrix Analysis

**Figure 4:**
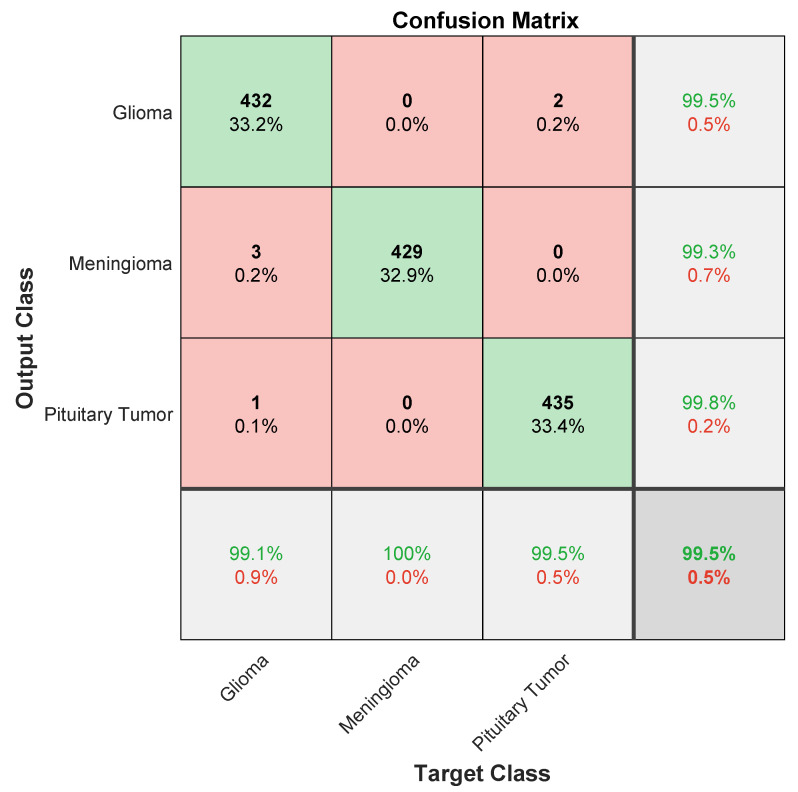
Confusion matrix analysis of ESRNet (Green indicates True Positives (correct predictions), while other colors represent errors, such as False Positives.).

Figure 4 presents the confusion matrix depicting the performance of ESRNet in BTC. Notably, glioma, meningioma, and pituitary tumors were all accurately identified, resulting in an impressive overall accuracy of approximately 99.5%. This underscores the model’s adeptness in correctly predicting instances for each specific class. The consistently high values for each class further affirm the robustness of ESRNet in achieving precise and reliable BTC.

### 4.5. Receiver Operating Characteristic (ROC) Curve Analysis

**Figure 5:**
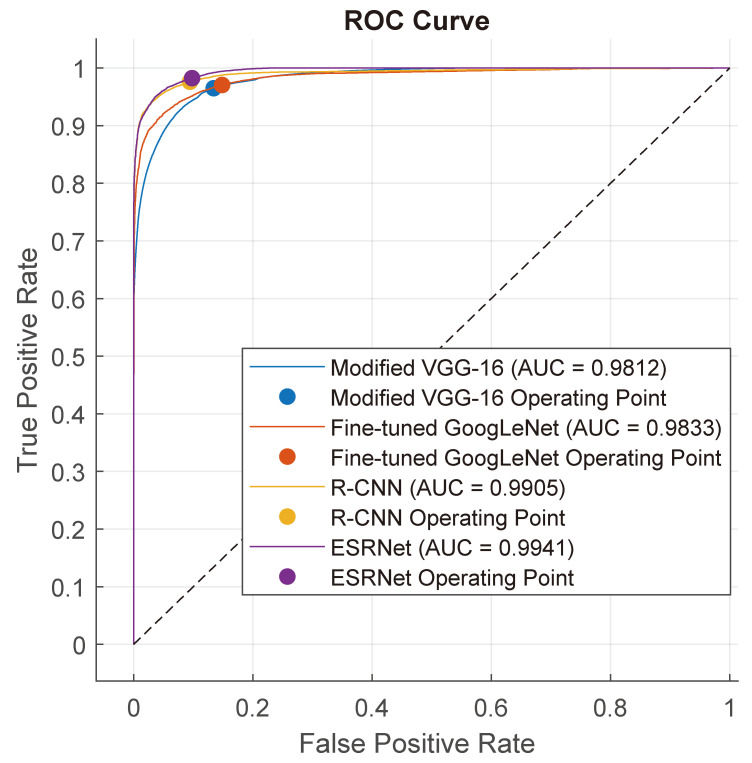
Receiver operating characteristic (ROC) curve analysis.

Figure 5 presents a ROC analysis of modified VGG-16, R-CNN, fine-tuned GoogLeNet, and the proposed ESRNet. It showcases the balance between the false positive rate (FPR) and the true positive rate (TPR) for each model. It demonstrates that the proposed ESRNet achieves significantly superior performance in terms of ROC, indicating its effectiveness in distinguishing between positive and negative cases. Importantly, the comment underscores the noteworthy achievement of the proposed ESRNet model, showcasing remarkable results with an area-under-the-curve (AUC) value of 0.9941.

### 4.6. Comparative Analysis

**Figure 6:**
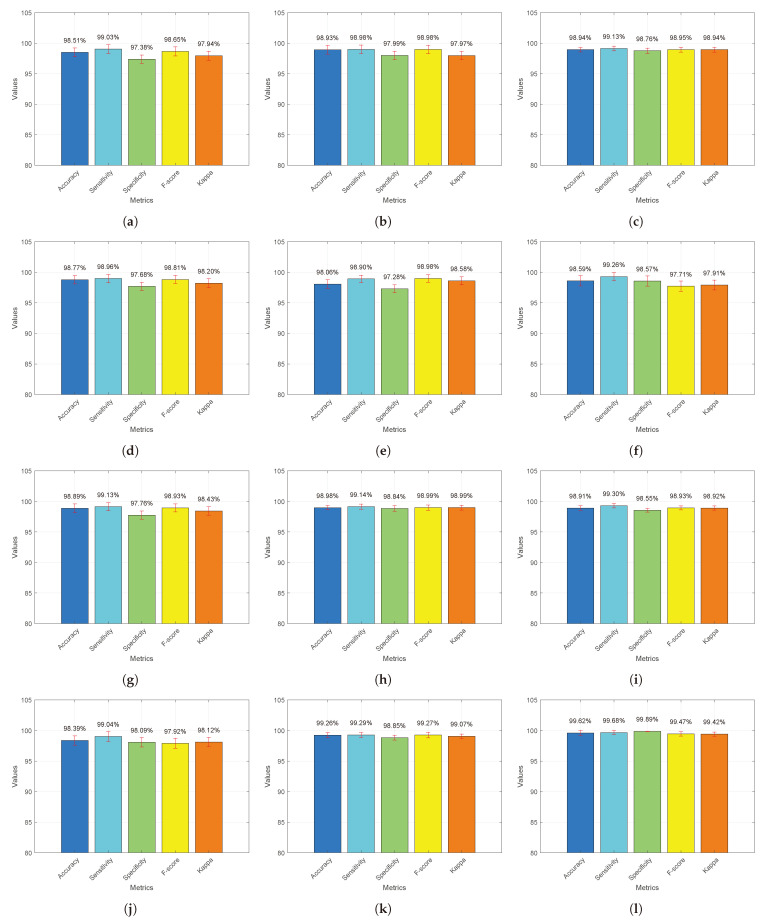
Comparative median analysis between ESRNet and competitive models. (**a**) CNN [15]; (**b**) multi-scale CNN [16]; (**c**) ResNet-18 [30]; (**d**) CNN and RCNN [31]; (**e**) VAE and GAN [18]; (**f**) EfficientNets [19]; (**g**) BTC-fCNN [28]; (**h**) InceptionResNetV2 [29]; (**i**) modified VGG-16 [27]; (**j**) R-CNN [20]; (**k**) fine-tuned GoogLeNet [17]; (**l**) the proposed ESRNet.

Figure 6 presents a comparative analysis of the median values for each model, including (a) CNN [15], (b) multi-scale CNN [16], (c) ResNet-18 [30], (d) CNN and RCNN [31], (e) VAE and GAN [18], (f) EfficientNets [19], (g) BTC-fCNN [28], (h) InceptionResNetV2 [29], (i) modified VGG-16 [27], (j) R-CNN [20], (k) fine-tuned GoogLeNet [17], and (l) the proposed ESRNet. These models were evaluated across five essential metrics: accuracy, sensitivity, specificity, F-score, and Kappa, which are represented by a distinct bar color, i.e., blue, cyan, green, yellow, and orange, respectively. The median values were computed based on 30 separate evaluations for each model. Additionally, red bars representing the standard deviation (σ) values are provided to illustrate the degree of performance variation among these models. Overall, DSRNet demonstrated superior performance compared to the competitive models, consistently achieving significantly higher results.

Table 2 provides an extensive performance assessment of various models on the BTC dataset. The table presents a comprehensive performance evaluation of various models on the BTC dataset, with ESRNet emerging as the standout performer. ESRNet attained the highest metrics across accuracy (99.62 ± 0.28), sensitivity (99.68 ± 0.21), specificity (99.89 ± 0.10), F-score (99.47 ± 0.43), and Kappa (99.42 ± 0.21), showcasing its exceptional efficacy in brain tumor classification. ResNet-50 and DenseNet-121 exhibited robust performances, excelling in accuracy and sensitivity. InceptionV3 demonstrated competitive results, particularly in accuracy (98.94 ± 0.56) and sensitivity (99.12 ± 0.58). DSCNet stood out with remarkable sensitivity (99.39 ± 0.53), emphasizing its strength in capturing intricate tumor patterns. EfficientNet showcased balanced performance, underscoring its effectiveness in brain tumor classification. CNN, though slightly trailing in the metrics, maintained respectable accuracy (95.59 ± 1.74) and sensitivity (96.97 ± 1.42). ResNet-101 and AlexNet achieved commendable results, enriching the diversity of the effective models for BTC.

Among the models examined, it wa found that ESRNet significantly outperformed the others in terms of accuracy, sensitivity, specificity, F-score, and Kappa statistics, with median values of 99.62%, 99.68%, 99.89%, 99.47%, and 99.42%, respectively. Moreover, the achieved minimum performance metrics, including accuracy (99.34%), sensitivity (99.47%), specificity (99.79%), F-score (99.04%), and Kappa statistics (99.21%), underscore the exceptional effectiveness of ESRNet for BTC. These consistent and high-performing results across diverse metrics establish ESRNet as a standout choice, demonstrating remarkable accuracy and robust performance in brain tumor classification.

## 5. Conclusions

This paper introduced an ESRNet, representing a significant advancement in the field of BTC. The use of residual blocks with skip connections played a crucial role in enhancing the gradient flow during training, thereby addressing the vanishing gradient problem commonly encountered in existing models. The architectural design of ESRNet involved multiple stages, each featuring an increasing number of residual blocks, which promoted feature learning and facilitated pattern recognition. In addition to its architectural innovations, ESRNet incorporated efficient downsampling techniques and stabilizing batch normalization layers, contributing to its overall robustness and reliability. Extensive experimental results consistently demonstrated ESRNet’s superiority, with outstanding performance metrics, including accuracy (99.62%), sensitivity (99.68%), specificity (99.89%), F-score (99.47%), and Kappa statistics (99.42%). Overall, ESRNet emerged as a robust and efficient framework for BTC, promising improved performance and efficiency in tackling the critical challenges within the domain of medical image analysis. Its potential impact on clinical diagnosis and treatment planning for individuals with brain tumors is noteworthy.

## Figures and Tables

**Table 1:** Hyperparameters for ESRNet.

Symbol	Full-Form	Description	Used Value
α	Learning Rate	Rate of parameter updates	0.001
*B*	Batch Size	Number of samples per mini-batch	32
$N_{\mathrm{epochs}}$	Number of Epochs	Total training epochs	700
*F*	Filter Count	Number of filters in conv. layers	64
$d_{1}$	Dropout Rate	Probability of neuron dropout 1	0.3
$d_{2}$	Dropout Rate	Probability of neuron dropout 2	0.2
*S*	Stride	Step size in conv. layers	1
$\beta_{1}$	First Moment Decay Rate	Decay rate for first moment estimates	0.9
$\beta_{2}$	Second Moment Decay Rate	Decay rate for second moment estimates	0.999
ϵ	Epsilon	Smoothing term in Adam optimizer	$1\times 10^{-7}$
Kernel Size	Convolution Kernel Size	Size of conv. kernels	3 × 3
Padding	Convolution Padding	Type of padding	‘same’

**Table 2:** Performance evaluation of competing and proposed models on BTC dataset.

Model	Accuracy	Sensitivity	Specificity	F-Score	Kappa
CNN	95.59 ± 1.74	96.97 ± 1.42	92.56 ± 1.65	95.61 ± 1.56	95.61 ± 1.70
ResNet-101	96.29 ± 1.94	98.04 ± 1.80	97.81 ± 1.87	96.43 ± 1.82	96.43 ± 1.86
ResNet-50	97.92 ± 1.36	96.98 ± 1.47	95.99 ± 1.37	97.98 ± 1.25	97.96 ± 1.22
AlexNet	96.89 ± 1.02	98.82 ± 0.87	97.67 ± 0.99	96.92 ± 0.99	96.92 ± 1.01
EfficientNet	98.07 ± 1.05	98.90 ± 0.92	96.38 ± 0.97	98.08 ± 0.94	98.08 ± 0.98
InceptionV3	98.94 ± 0.56	99.12 ± 0.58	98.67 ± 0.72	98.95 ± 0.70	98.94 ± 0.59
ESRNet with SGD	98.91 ± 0.56	99.20 ± 0.54	98.55 ± 0.45	98.92 ± 0.49	98.93 ± 0.53
DSCNet	99.07 ± 0.54	99.39 ± 0.53	98.85 ± 0.41	99.06 ± 0.49	99.06 ± 0.54
ESRNet	**99.62 ± 0.28**	**99.68 ± 0.21**	**99.89 ± 0.10**	**99.47 ± 0.43**	**99.42 ± 0.21**

## Data Availability

The dataset is freely available at https://figshare.com/articles/dataset/brain_tumor_dataset/1512427 and https://www.kaggle.com/datasets/obulisainaren/multi-cancer.
